# Supplementary material for: Designing PrEP and early HIV treatment interventions for implementation among female sex workers in South Africa: developing and learning from a formative research process
Source: BMJ Open. 2018 Jun 14;8(6):e019292. doi: 10.1136/bmjopen-2017-019292 (PMC6009452; doi:10.1136/bmjopen-2017-019292)
Supplement: Author's manuscript [file bmjopen-2017-019292.draft_revisions.pdf]

BMJ Open is committed to open peer review. As part of this commitment we make the peer review history of every article we publish publicly available.

When an article is published we post the peer reviewers' comments and the authors' responses online. We also post the versions of the paper that were used during peer review. These are the versions that the peer review comments apply to.

The versions of the paper that follow are the versions that were submitted during the peer review process. They are not the versions of record or the final published versions. They should not be cited or distributed as the published version of this manuscript.

BMJ Open is an open access journal and the full, final, typeset and author-corrected version of record of the manuscript is available on our site with no access controls, subscription charges or pay-per-view fees (http://bmjopen.bmj.com).

If you have any questions on BMJ Open's open peer review process please email info.bmjopen@bmj.com

BMJ Open

Designing PrEP and early HIV treatment interventions for implementation among female sex workers in South Africa: developing and learning from a formative research process

Journal:	*BMJ Open*
Manuscript ID	bmjopen-2017-019292
Article Type:	Research
Date Submitted by the Author:	29-Aug-2017
Complete List of Authors:	Eakle, Robyn; Wits Reproductive Health and HIV Institute, ; London School of Hygiene and Tropical Medicine, Social and Environmental Health Research<br>Mutanha, Nyaradzo; Wits Reproductive Health and HIV Institute<br>Mbogua, Judie<br>Sibanyoni, Maria ; Wits Reproductive Health and HIV Institute<br>Bourne, Adam; La Trobe University, Australian Research Centre for Sex, Health & Society<br>Gomez, Gabriela; London School of Hygiene and Tropical Medicine,<br>Venter, WD Francois; University of the Witwatersrand, Wits RHI<br>Rees, Helen; University of the Witwatersrand, Wits RHI
Keywords:	Sex work, HIV prevention, pre-exposure prophylaxis, formative research

SCHOLARONE™ Manuscripts

For peer review only

Designing PrEP and early HIV treatment interventions for implementation among female sex workers in South Africa: developing and learning from a formative research process

Robyn Eakle*[1, 2,3], Nyaradzo Mutanha[1], Judie Mbogua[1], Maria Sibanyoni[1], Adam Bourne[4], Gabriela Gomez[1, 2], Francois Venter[1], Helen Rees[1]

[1]Wits Reproductive Health and HIV Institute, Faculty of Health Sciences, University of the Witwatersrand, Johannesburg, South Africa
[2] Social and Mathematical Epidemiology (SaME), Department of Global Health and Development London School of Hygiene and Tropical Medicine, UK
[3] Sigma Research, Department of Social and Environmental Health Research, London School of Hygiene and Tropical Medicine, UK
[4] Australian Research Centre for Sex, Health & Society, La Trobe University, Melbourne, Australia.

*Corresponding author:
Robyn Eakle
Wits Reproductive Health & HIV Institute
University of the Witwatersrand,
Hillbrow Health Precinct
22 Esselen Street
Hillbrow, 2001
Johannesburg
South Africa
e: reakle@wrhi.ac.za

Author emails:

Robyn Eakle	robyn.eakle@lshtm.ac.uk; reakle@wrhi.ac.za
Nyaradzo Mutanha	missnyarie@gmail.com
Judie Mbogua	judie.mbogua@gmail.com
Maria Sibanyoni	msibanyoni@wrhi.ac.za
Adam Bourne	A.Bourne@latrobe.edu.au
Gabriela Gomez	Gabriela.Gomez@lshtm.ac.uk
Francois Venter	fventer@wrhi.ac.za
Helen Rees	hrees@wrhi.ac.za

Word count: 5,446
Number of figures/tables: 1
References: 25

Key words: HIV prevention, biomedical prevention products, pre-exposure prophylaxis (PrEP), sex work, formative research

For peer review only - http://bmjopen.bmj.com/site/about/guidelines.xhtml

For peer review only

Abstract

Objectives

This paper examines how an inductive approach, based on the principles of grounded theory, to formative research influenced the design and execution of oral pre-exposure prophylaxis (PrEP) and early antiretroviral (ART) interventions for female sex workers (FSWs) in South Africa.

Setting

The formative research was carried out in a variety of locations as dictated by the inductive approach. These included five sites in and around sex worker clinics, and at stakeholder offices.

Participants

Participants in this research included stakeholders, experts in the field, and FSWs. Since this was an evolving, inductive approach to research, numbers of participants were not always tabulated except in the case of focus group discussions (FGDs).

Results

Results consisted of methods chosen and subsequent data. Five chosen methods were: 1) stakeholder consultations launching and evolving the research; 2) engagement with FSW communities and working environments including hotspot mapping and clinic interactions; 3) site feasibility assessments for site selection; 4) supportive structures development for retention and adherence; 5) FGDs conducted with FSWs to explore specifics of acceptability. This process identified implementation barriers, opportunities, and research gaps critical to intervention design. Two sites were selected in Johannesburg and Pretoria, out of five considered. The contexts of the two urban sites varied, necessitating adjustments to intervention implementation.

Conclusions

Using an inductive approach allowed for a wide range of perspectives, defining population needs and how to best reach them. This research illustrated how similar sex work environments can vary and how implementation of interventions may not be uniform across contexts. Lessons learned in details could assist in future project designs and implementation of new interventions where feasibility, social and cultural factors affecting acceptability must be considered.

Strengths and Limitations

- This formative research process drew on principles of grounded theory allowing for an inductive, iterative approach to drive the selection of the most appropriate methods for gathering a broad spectrum of data aimed at intervention design.
- Five components were selected through the research process, providing an array of data for decision making and intervention design.
- This was a systematic approach in five known sex worker sites and final sites were chosen for their nearer uniformity than for diversity; results may not translate beyond this study, however lessons learned will be applicable.

For peer review only - http://bmjopen.bmj.com/site/about/guidelines.xhtml

For peer review only

Introduction

Several guidance documents highlight the need for formative research both when preparing for larger studies and to design the implementation of new public health HIV interventions (1,2). Formative research includes the assessment of feasibility, reach, acceptability, and need of populations to strengthen planned uptake and use of interventions. In particular, formative research aims to ensure the capacity for physical implementation and responsiveness to cultural, social, economic and physical environments (3–5). However, this phase of work is frequently not reported and important lessons learned may be lost. This paper describes the detailed decision making and conduct of formative research undertaken to design two new HIV prevention and treatment interventions delivered to female sex workers in a demonstration project in South Africa.

Oral pre-exposure prophylaxis (PrEP) using antiretroviral (ARV) drugs given to an HIV-negative individual to prevent HIV infection, has been shown to be efficacious in multiple clinical trials (6). In additional, HIV treatment can be given to HIV-positive people as soon as they are diagnosed, and together with oral PrEP, is now the standard of care recommended in 2015 by the World Health Organization (WHO) (7).

Demonstration projects were recommended by the WHO in 2013 to generate evidence to answer implementation questions around feasibility and acceptability of oral PrEP (8). The call prioritised research for key populations such as sex workers, who have been shown by modelling to be ideal candidates for PrEP, especially in combination with early antiretroviral (ART) treatment for HIV-positive people (9–11). In the previous decade, HIV prevalence among female sex workers in South Africa was found to be between 46% and 69% (12–14), with recent research estimating a prevalence of 72% in the greater Johannesburg area (15).

This paper examines and illustrates how a comprehensive and inductive approach to formative research based on grounded theory (16) informed the design and execution of oral PrEP and early antiretroviral (ART) interventions for female sex workers in South Africa as part of the TAPS (Treatment And Prevention for Sex workers) Demonstration Project. The purpose of TAPS was to demonstrate how these two interventions could be implemented among female sex workers, and inform national rollout. We explore the approach and process undertaken to define and carry out the formative research, describe how the results informed the overall design of the oral PrEP and early ART interventions for TAPS, and reflect on challenges and successes encountered during the process.

Methods

Formative research can include an array of methods depending on the final desired outcomes. For the design of interventions, formative research will include exploring feasibility of supportive structures and logistics for physical delivery of the intervention, as well as exploring the acceptability among populations in different contexts (2).

The overall aims of the larger TAPS Demonstration Project were to explore whether FSWs will take up early ART or PrEP, whether the service delivery mechanism is capable of handling the increase in

For peer review only - http://bmjopen.bmj.com/site/about/guidelines.xhtml

resource needs that might be required, and what the implications of the implementation of these interventions might be, including overall costs should the interventions be scaled up (17). TAPS is part of the Wits RHI Sex Worker Programme (SWP), a comprehensive health and well-being programme for sex workers running for over 20 years in Johannesburg and other provinces in South Africa (18).

While TAPS itself aimed to answer questions of feasibility and acceptability in the actual delivery of PrEP and early ART for female sex workers, the formative research began with foundational concepts of feasibility and acceptability in order to design interventions which could then be evaluated. These concepts addressed questions including: whether there was initial support from stakeholders to test the implementation of PrEP and early ART; where could the interventions be delivered, how, and by whom; how to engage with female sex workers; what was needed to generate demand; what structures should be included to support delivery; and how did women conceive of acceptability as users of the interventions?

The formative research was conducted between August 2013 and March 2015. An inductive approach was employed based on the principles of grounded theory (16), where lessons emerging from the data at each step in the research process dictated decisions and subsequent steps. A grounded approach was chosen given the wide original scope required to consider a large range of logistical possibilities (e.g. site locations, adherence support structures) for the design and implementation of the interventions.

Decision making about which methods, sites, and stakeholders with whom to engage at each step was guided by feedback from consultations and discussions. Principles from the Good Participatory Practice Guidelines (GPP) developed by UNAIDS and AVAC were also followed, promoting multi-level stakeholder engagement as a core component of research (1). In line with these guidelines, a range of stakeholders were engaged, including sex workers, sex work related organizations, and the Department of Health (DoH). Community mobilization and outreach served to develop awareness around the interventions and generate demand led by peer educators who were current sex workers. Site assessments were conducted as part of feasibility which included site visits, and took into account findings from the community engagement and 'hot spot' mapping activities (identifying areas in which multiple sex workers operate). Additionally, focus group discussions (FGDs) were held with female sex workers at the final selected sites. Since the development of the methods for this process represents results in and of themselves, the specifics of how activities were chosen, why, and what was learned, are detailed as part of the results.

These activities eventually fell into five core methods: consultations, community engagement and mapping in the field, site assessments, participatory development of supportive structures and messaging, and FDGs. These methods generated three primary sources of data which informed the design of the interventions: 1) recorded minutes and reports of meetings with stakeholders; 2) field notes from engaging with sex worker communities and the environments within which they work, the process of hotspot mapping, experiences at potential clinic sites, and through the development of supportive structures; and, 3) transcripts of FGDs. Outcomes included the final design of the intervention incorporating perspectives of stakeholders gathered from the data, as well as relevant

For peer review only - http://bmjopen.bmj.com/site/about/guidelines.xhtml

data collection and monitoring tools and supportive structures. These data were collected by a combination of researchers, peer educators, and clinical staff.

Data analysis

Data were continuously collected and analysed based on the inductive approach over 18 months. Field notes, meeting notes, written reports, and FGD transcripts, were reviewed as activities occurred to identify key themes for further exploration and to define next steps, such as whether to disqualify a site due to low accessibility of the population or where to spend more time on community mobilization, education, and hotspot mapping. Site selection and staff recruitment represented the end of the first major phase of formative research. Community mobilization was then focused at the selected sites where testing of messaging, development of supportive systems, and development and testing of potential data collection tools continued led by clinic staff, peer educators, and potential end users.

FGDs were conducted at each of the final selected sites in multiple languages to suit the participants and analysed following principles of thematic analysis (19), concentrating on themes originating from the FGD guides and those emerging from the discussions. Further details of the methods and results from the FGDs are presented in a companion paper (20).

Results

Results are presented in five sections according to the chosen methods as products of the grounded approach. Sections include how and why methods were chosen and undertaken, and lessons learned influencing the design and execution of the interventions.

The five methods were: 1) stakeholder consultations both launching and iteratively progressing the research with stakeholder perspectives; 2) community engagement in the field with sex workers and the environments within which they work including hotspot mapping and experiences at potential clinic sites; 3) site assessments to determine feasibility of delivering the interventions in given sites; 4) development of supportive structures to encourage retention and intervention adherence; 5) FGDs conducted with potential end-users to explore specifics of acceptability. A diagram of the five methods and the timeline during which they occurred is shown in Figure 1, illustrating how some activities were continuous and others discrete.

[Insert Figure 1. Formative Research Process Graphic]

Stakeholder Consultations

Research began with this activity in order to ascertain stakeholder knowledge of and attitudes towards PrEP and early ART, as well as attitudes towards sex workers, which denoted initial levels of acceptability for the interventions in this population. We conducted three community consultations in 2013 with a total of 81 attendees from sex worker communities and partner organizations in Hillbrow and Ngodwana, where a new sex worker clinic was in the initiation phase. Additionally, an international consultation of 38 attendees was held in Hillbrow in 2013 and included representatives from sex work communities and organizations, funders, UNAIDS and WHO (21). These consultations

For peer review only - http://bmjopen.bmj.com/site/about/guidelines.xhtml

allowed for mapping of organizations and individuals to be included in future consultations and phases of formative research.

Smaller meetings were held with local DoH representatives in Johannesburg, Mpumalanga, Phongolo, and Pretoria to develop partnerships and support agreements for the TAPS project. Updates on, and engagement with, the plans for the TAPS interventions continued at quarterly and other scheduled meetings with local DoH and partners. Parallel meetings were also held with National DoH and the South African National AIDS Council (SANAC).

Feedback from the consultations pointed to varying degrees of support depending on local capacity to take on new interventions and scepticism towards the interventions, and particularly PrEP. Stakeholders affirmed that implementing PrEP and early ART together would promote synergistic delivery, as well as potentially normalize the use of ARVs in providing options to both HIV-negative and positive sex workers. However, concerns were voiced about adherence to PrEP (and sometimes early ART), as well as the potential for reduction in condom use, increases in risk of resistance to ARVs, and burden on scarce resources. These concerns indicated where special attention should be focused, such as providing evidence about possible resistance to PrEP and strategies for and the philosophy around supporting adherence and condom use, both in early messaging and education for sex workers, implementing and policy partners, as well as where to focus messaging and monitoring when implementing the interventions in TAPS.

Sex workers at each potential site, driven largely by peer educators, helped to identify community organizations to engage for additional perspectives. Stakeholder engagement, both formal and informal, was tracked using an engagement tracking tool (22) developed by the HIV prevention advocacy group, AVAC. According to the data recorded in the tool, at least 20 meetings occurred during this time; however it is not possible to definitively quantify all of the contact points given that many interactions occurred on an ad hoc basis.

Finally, consultations were also used to identify potential community advisory board (CAB) members. Meetings were held with individuals expressing interest in participation to inform them about the study and the CAB. These discussions continued until a CAB with up to 12 members was established. The CAB incorporated representatives from the local police force, sex worker advocacy organizations, sex workers themselves, and religious organizations. The CAB became both a result of the formative research and another source of consultation. The CAB was also a supportive structure for the interventions as members took it upon themselves to help spread knowledge of the interventions and the TAPS project, as well as lobby for expansion of the interventions to more sites, populations, and organizations.

Community Engagement and Mapping

Data were also collected through community mobilization activities, accruing organically in scope and number, as discussions and interactions within the communities pointed to additional contacts for engagement. Each point of contact presented opportunities for new consultations and/or locations to conduct outreach. Feedback from the community about potentially adding PrEP and early treatment as new HIV prevention and treatment options was collected during peer and clinical

For peer review only - http://bmjopen.bmj.com/site/about/guidelines.xhtml

staff outreach and workshops, which also aimed to generate demand for the interventions in the potential demonstration project site communities.

Outreach is a specific set of activities in the sex worker service delivery field undertaken to educate clients about health, promote condom use, and provide a spectrum of services from HIV testing and counselling to full clinical services in mobile units in locations accessible to the population (23). Staff and peer educators from the SWP, in collaboration with TAPS staff and researchers, conducted outreach as a team to probe the local sex worker populations for any knowledge or perceptions of PrEP and early ART, as well as start to introduce related information. This outreach occurred as part of existing programming consisting of health talks, condom distribution, and mobile clinic services, through which we progressively integrated education and awareness of PrEP and early ART. As a core feature of the SWP, interventions were designed around outreach which was identified as a central aspect critical to generating demand and recruiting participants for TAPS. Through this activity, we noted working hours and environmental contexts, created hot spot maps to focus recruitment efforts, and learned about community perspectives of PrEP and early ART.

Feedback collected during outreach in the form of field notes provided contextual information as to where and how sex workers operated and how they interacted with health services, informing viability of given sites. Cursory understanding of the interventions and how they might be taken up and used, determined which supportive structures would be needed and how messages would have to define the differences between PrEP and early ART. These outcomes are described in further sections.

Site assessments and selection

Assessments were conducted in five sites, after eliminating other sites spanning multiple provinces in the Wits RHI SWP. The five sites featured existing SWP clinics already delivering ARVs per national guidelines or sites with plans to implement new clinics, interest and support from the local communities, and potential access to a large group of FSWs in areas where HIV prevalence was high. This information came from the initial consultations and community engagement and mapping, as well as internal programme data. The five sites were: Ngodwana (rural village in Mpumalanga province), Phongolo (rural trucking site in KwaZulu Natal province), Hillbrow (central, inner-city Johannesburg), City Deep (peri-urban trucking site immediately south of Johannesburg), and the Pretoria central business district (CBD), the latter three of which were located in Gauteng province.

Narrowing the site selection was a critical step in moving forward with the design of the interventions. We aimed to select 2-3 sites (according to funding and capacity for oversight) with the following population criteria: access to a large number of FSWs (>200 according to early sample size calculations (17)), populations with a relatively balanced proportion of HIV-negative and HIV-positive women, and accessibility of clinics. Physical feasibility of the clinics to implement the interventions was assessed through reviews of space, clinical and laboratory infrastructure, required site permissions and approvals, and supply chain mechanisms. Support for the interventions was explored with local DoH and sex worker communities, as well as identification of logistical gaps. Site visits, hot spot mapping, and site assessments were conducted to determine the feasibility of delivering PrEP and early ART in each of the five sites.

For peer review only - http://bmjopen.bmj.com/site/about/guidelines.xhtml

Hot spot mapping was conducted to determine the number of FSWs working near the potential study sites as well as their working hours. The methods utilized and built on the results produced by the South African Health Monitoring Study (SAHMS), which drew on multiple mapping methods including time-location and the “wisdom-of-crowds” (15). This activity in combination with prior experience derived through the SWP, revealed that time and location of sex work is driven by client availability. Peak working hours were recorded in each sites as critical data dictating when women could be free to attend the clinics. Local languages at each site were also documented so that study materials could be translated and appropriate staff hired.

Following the site assessments, three sites were eliminated. Ngodwana was eliminated due to lack of infrastructure and building delays, as well as lack of local support. Although there was initial interest, the implementation of the interventions potentially conflicted with limited resources and competing priorities in the area. The rural sex worker community in that location do not self-identify as sex workers which would have created challenges in being able to compare formal sex workers who self-identify and those who do not as part of project evaluation. The prevalence of HIV in Ngodwana was estimated locally to be around 75-80% in a relatively small village of <300 people mostly made up of sex workers, which also would have made it difficult to implement PrEP.

City Deep was eliminated due to low clinic attendance, as well as the highly transient nature of the sex worker populations. Most of the women attending that clinic in arrived in the trucks coming from all over South Africa as well as neighbouring countries, which would have made commitment to regular, repeat clinic visits difficult. The Phongolo site, located next to the Swaziland border, was also eliminated as women tended to move in and out of the town and were away for as many as three to four months at a time.

Two sites were finally selected: Hillbrow and Pretoria. Hillbrow was chosen because of the long-standing Wits RHI sex worker clinic which maintains strong ties to a large community of sex workers and their managers (brothel owners, ‘pimps’). At the time, HIV prevalence was estimated locally at around 50%. The clinic is situated in an area with a high concentration of brothels and street based locations within walking distance, but also with a number of surrounding outreach areas in the Johannesburg CBD, Yeoville, Jeppe, and Rosettenville. For these reasons, this site was considered “low-hanging fruit” for new intervention implementation, due to the substantial population of women at risk of HIV infection and as a location with solid community support.

In Pretoria, tailored sex worker services and relationships with the community were new at the Sediba Hope clinic in the heart of the CBD, however there was strong support from the local DoH and expressed need from the sex workers themselves. The newness of such interventions in the area posed a challenge as to whether PrEP and early ART could be implemented within a new sex worker clinic in a new setting, and what it would take to do so.

After final site selection, further identifying and understanding the contexts in which the women worked and lived, as well as their perspectives of those environments, was explored through more intensive outreach and community engagement to determine, on a granular level, how the interventions could fit into their lives. Through the existing, long-term work of the SWP, there was awareness of different types of sex work locales, however the formative research allowed for a

For peer review only - http://bmjopen.bmj.com/site/about/guidelines.xhtml

deeper understanding of how these locales affected women's lives, and how they might affect their ability to take up and use the interventions.

We assumed three general categories of sex work locales within the two urban settings. Brothels or hotels usually offer alcohol and entertainment, including combinations of music, dancing, and bar games. Many are former or existing hotels while others are simply bars with backrooms. Security protecting sex workers from violent clients is common in these establishments. Secondly, street-based sex work occurs in high traffic areas where sex workers pick up clients, and either join them in vehicles or have sex in a nearby location such as an alley. The third category comprises the "dark places" (IsiZulu: *mnyamandawo*), informal locations in empty lots or other uninhabited/unoccupied buildings or areas where sex workers build rudimentary structures in which to work.

In both cities there is also a fourth, more hidden sex work market found online. Awareness of the online market was generated through conversations with sex workers during outreach and through peer educators, some of whom had online pages and knew of others with similar arrangements. Sex work conducted online usually involves regular clients met through a web-based connection, where business is conducted out of private spaces (personal homes, private spaces run by an owner, upscale hotels or clubs). This population was engaged later in the process as they are less visible to implementers and researchers, and difficult to build relationships with given their desire for anonymity.

The different types of locales dictated how outreach needed to be conducted in order to generate demand for the interventions. In brothels it was possible to hold group health talks in spaces lent by women or brothel managers, but engaging with women on the street or dark places required time to build trust and a certain level of discretion to avoid unwanted attention. Careful planning around space needs (setting up a rented or borrowed space for sex workers based on the street, or bringing a mobile clinic) was also necessary in these locales. Similarly, it was not possible to go to a place of work to conduct health talks and recruitment for participants identified online, rather relationships were developed with one or two women who could act as advocates for the interventions within their community.

The different risks and benefits for sex workers in each locale type were important to take into account. In brothels, women are protected by managers, staff, and security. On the street and "dark places" there is little or no security, unless organised by a pimp or a gang. Stories of violence and rape were commonly recorded throughout the formative work. Women reported violent interactions with clients, managers, community members, and often the police. These interactions highlighted the need for supportive referral structures, including for mental health, serious injuries, and criminal issues.

Earning ability, directly associated with the type of sex work locale, was identified as an important aspect of being able to take time off to come to the clinic even for short periods of time. Sex workers operating in brothels tend to be able to charge higher rates for sex, usually between R50 and R150 (about 3-10 USD), depending on the status of the brothel and the cost of room rental incurred by the sex workers. On the street, rates are around R50, and in a dark place sex can be R20-30 (about 1.30-

For peer review only - http://bmjopen.bmj.com/site/about/guidelines.xhtml

2 USD). Making less money meant more difficulty in taking time away from work to attend the clinic for regular PrEP or treatment related appointments.

Development of supportive structures and messaging

During consultations, a number of stakeholders provided views on possible components to be incorporated in the intervention design, in particular supportive structures. Mechanisms for supporting intervention adherence and retention in care were explored, however we decided only to consider those which could also be incorporated into a national programme. Among the possibilities were the use of MEMS caps (electronic bottle caps which would count bottle openings as a proxy for pill withdrawals and thus, adherence), pill counts (as conducted in clinical trials), and mHealth solutions in the form of short message service (SMS) messages. After discussing options with DoH partners, it was concluded that SMS as demonstrated by the ongoing MAMA Connect project (24), could be scaled up in a national programme, whereas other options would not be feasible.

A participatory process led by representatives of RHI's mHealth and Mental Health teams was undertaken through a series of workshops where themes around healthy living were developed followed by relevant SMS messages by peer educators. Both male and female sex worker peer educators worked in groups to create the messages which aimed to encourage healthy choices and wellness. These messages took into account the importance of avoiding inadvertent disclosure of HIV status to non-participants, and could still be used by the SWP after TAPS concluded. This process produced 110 SMS messages which would be sent once a week in succession to all participants who signed up for the service.

Experiences from early outreach activities directly influenced the messaging employed in each location, as well as educational materials used as part of supportive structures for generating demand and promoting awareness of the interventions. Messages focused on defining the interventions, addressing common myths and concerns around side effects, and providing information on efficacy and access.

Peer educator and potential user feedback indicated that personal testimonies are highly valued by women in both locations, so these were included on the informational pamphlets. Trained peer educators also enrolled in the study were invited to become ambassadors for the interventions so that they could directly relate their experiences and dispel rumours about side effects.

Finally, additional supportive structures for the interventions and the women using them included the CAB, as well as tried and tested referral systems where we could ensure women would get additional support beyond the scope of our clinics as needed. One result of the consultations, as well, was the need for holistic sensitivity training around sex work which was conducted with every staff member, from cleaner to clinician, at both sites. Although a more passive support mechanism provided by a local community partner organisation, this was important in ensuring women felt welcome at the clinics.

For peer review only - http://bmjopen.bmj.com/site/about/guidelines.xhtml

Focus Group Discussions

FGDs were conducted in the Hillbrow and Pretoria CBD sites following site selection as the final set of activities in the formative research process. The FGDs aimed to test the core intervention design components for acceptability among potential users within the target communities. Four FGDs were held in each site with a total of 69 participants. The FGDs comprised important final steps in informing design as they explored a more focused community perspective of intervention acceptability on two main levels. One level consisted of data concerning logistics and preferences around physical delivery (location, preferred clinic times, frequency of visits and HIV testing), while the other level included social and structural level data where elements of stigma and socioeconomic norms (e.g. where the need for income might supersede health) that might affect uptake and use of PrEP and early ART were explored. Since this paper is focused on feasibility and early stages of acceptability, the end-stage formative data from the FGDs are presented in a companion paper (25).

Discussion

In this paper, we have described in detail the formative research process and findings used to inform the design of the PrEP and early ART interventions implemented in TAPS. The inductive approach afforded a breadth of information around potential site locations, community and stakeholder perspectives, including potential barriers to successful implementation, and nuanced aspects of the urban sex work settings for considering how to ensure the interventions accomplished reach, accessibility, acceptability, and filled the needs of potential end-users. This process allowed for rapid and relevant consolidation of lessons learned to inform adaptations to the planned interventions.

Feasibility played a significant role in early decision making as to whether PrEP and early ART could be implemented, and acceptability from stakeholders ran in parallel. Questions of feasibility addressed site capacity, experience in delivering ARVs, experience with and access to FSWs, site locations related to FSW populations, and existing supply chains. Early acceptability was determined through consultations and engagement with potential end users in the community during outreach. Factors influencing feasibility and acceptability would not have been as comprehensively understood, such as the day to day clinic operation and the physical contexts and locales of sex work, without repeated site visits and time spent in the field. Continuous, in-depth interactions with the women themselves allowed the TAPS team to better understand sex workers' needs in addition to how interventions reach could be maximized. Additionally, identifying and addressing potential biases from providers and other stakeholders in the provision of the interventions was essential to avoid issues with maintaining permissions and supply chains as well as supportive services.

Generally, it would be expected that sex work populations and industries, with similar urban contexts and in relative geographical proximity, would be the same in both places and that interventions could be implemented uniformly in both locations. However, the formative research demonstrated just the opposite. While there were some similarities in how women live and work in terms of the types of locales and spaces, there were also significant differences. Sex work locales can be similarly categorized in the two cities, but the organization of these spaces and the make-up of them within Hillbrow and Pretoria is quite different in terms of women's personal safety and earning capability, for example. This translates into different sex worker populations and market dynamics and the need for adaptations to intervention design. Outreach strategies (groups versus one on one

For peer review only - http://bmjopen.bmj.com/site/about/guidelines.xhtml

discussions) and messaging channels (word of mouth, top down through brothel owners and pimps, or developing online contacts) were dictated directly by context and the expressed needs of the FSWs.

The formative research created awareness for the TAPS team around which stakeholders needed to be involved and engaged throughout the duration of the project, either through the CAB or in regular consultations, as well as the contexts in which women worked and lived and the need to be sensitive around FSW engagement to generate demand for PrEP and early treatment. Without the formative research, we would not have understood the importance of some messages over others from the perspectives of sex workers, the dynamics of sex work locales and their effects on risk and income, nor how flexibility around clinic visit scheduling would need to be taken into account.

Conclusions

The lessons learned from this formative research process were directly applied to the design and implementation of the PrEP and early ART interventions delivered through the TAPS project. The inductive approach afforded the opportunity to adapt and include voices and perspectives, which might have otherwise been missed, and clarified the needs of the population as well as how to reach them appropriately. This research illustrated how sex work environments can vary, even when the settings are very similar, and therefore implementation of interventions is unlikely to be uniform across contexts. Formative research is critical in designing interventions, especially in new environments but also in well-known contexts. Including intensive stakeholder engagement in formative research will help to ensure that interventions are designed with feasibility and relevance for populations in mind.

Declarations

Ethics approval and consent to participate

Formal ethical approval was provided by the Wits Human Ethics Research Committee (reference number: M131009) for the focus group discussions which included information testing and community engagement in the field to inform the development of discussion guides. All documents containing data were saved electronically in central folders with limited access to relevant project staff. No participant identifiers were included in any of the reports that were produced. With regard to other formative research elements, no individual data were collected or reported, and ethical principles of the Helsinki Declaration were strictly adhered to as part of this iterative and flexible approach.

Availability of data and materials

All data generated or analysed during this study are included in this published article.

Competing interests

The authors have no declarations or conflicts of interest associated with this work.

Funding

This research was in part supported by funds from AIDS Fonds Netherlands (and the Bill and Melinda Gates Foundation (grant OPP1084416).

For peer review only - http://bmjopen.bmj.com/site/about/guidelines.xhtml

Author Contributions

RE developed the research agenda, designed data collection tools, participated in data collection, wrote and collected field notes, analysed the data, and drafted the paper. JM participated in data collection and analysed data. NM and MS participated in data collection. All authors reviewed and contributed to the paper.

Acknowledgements

We would like to thank the many women who participated in this formative research as well as the stakeholders and staff who engaged in helping us design the PrEP and early ART interventions for the TAPS Demonstration Project.

For peer review only

For peer review only - http://bmjopen.bmj.com/site/about/guidelines.xhtml

References

1. UNAIDS. Good participatory practice: Guidelines for biomedical HIV prevention trials [Internet]. 2011 [cited 2016 Sep 28]. Available from: http://www.unaids.org/en/resources/documents/2011/20110629_JC1853_GPP_Guidelines_2011%20OK

2. Tolley EE, Ulin PR, Mack N, Succop SM, Robinson ET. Qualitative Methods in Public Health: A Field Guide for Applied Research. John Wiley & Sons; 2016. 480 p.

3. Milat AJ, King L, Bauman AE, Redman S. The concept of scalability: increasing the scale and potential adoption of health promotion interventions into policy and practice. Health Promot Int. 2013 Sep 1;28(3):285–98.

4. Milat AJ, Bauman A, Redman S. Narrative review of models and success factors for scaling up public health interventions. Implement Sci. 2015;10:113.

5. World Health Organization. Scaling up projects and initiatives for better health: from concepts to practice [Internet]. 2016 [cited 2017 Apr 17]. Available from: http://www.euro.who.int/__data/assets/pdf_file/0004/318982/Scaling-up-reports-projects-concepts-practice.pdf

6. Fonner VA, Dalglish SL, Kennedy CE, Baggaley R, O'Reilly KR, Koechlin FM, et al. Effectiveness and safety of oral HIV preexposure prophylaxis for all populations. AIDS Lond Engl. 2016 Jul 31;30(12):1973–83.

7. World Health Organization. WHO | Guideline on when to start antiretroviral therapy and on pre-exposure prophylaxis for HIV [Internet]. 2015 [cited 2015 Oct 28]. Available from: http://www.who.int/hiv/pub/guidelines/earlyrelease-arv/en/

8. World Health Organization. PrEP demonstration projects: A framework for country level protocol development. A draft for review and discussion. 2013.

9. Cremin I, Alsallaq R, Dybul M, Piot P, Garnett G, Hallett TB. The new role of antiretrovirals in combination HIV prevention: a mathematical modelling analysis. AIDS. 2013 Jan;27(3):447–58.

10. Gomez GB, Borquez A, Case KK, Wheelock A, Vassall A, Hankins C. The Cost and Impact of Scaling Up Pre-exposure Prophylaxis for HIV Prevention: A Systematic Review of Cost-Effectiveness Modelling Studies. PLoS Med. 2013 Mar 12;10(3):e1001401.

11. Hallett TB. Priorities across the continuum of care: modelling the impact of interventions on mortality and HIV transmission. In Vancouver, Canada; 2015.

12. Rees H, Beksinska ME, Dickson-Tetteh K, Ballard RC, Htun Y. Commercial sex workers in Johannesburg: risk behaviour and HIV status. South Afr J Sci. 2000 Jun;96:283–4.

13. Williams BG, Taljaard D, Campbell CM, Gouws E, Ndhlovu L, Van Dam J, et al. Changing patterns of knowledge, reported behaviour and sexually transmitted infections in a South African gold mining community. AIDS Lond Engl. 2003 Sep 26;17(14):2099–107.

14. Ramjee G, Karim SS, Sturm AW. Sexually transmitted infections among sex workers in KwaZulu-Natal, South Africa. Sex Transm Dis. 1998 Aug;25(7):346–9.

For peer review only

For peer review only - http://bmjopen.bmj.com/site/about/guidelines.xhtml

15. UCSF, Anova Health Institute, Wits RHI. South African Health Monitoring Survey (SAHMS): An Integrated Biological and Behavioural Survey among Female Sex Workers, South Africa 2013 – 2014. San Francisco: UCSF; 2015.

16. The Discovery of Grounded Theory; Strategies for Qualitative... : Nursing Research [Internet]. [cited 2014 Jun 2]. Available from: http://journals.lww.com/nursingresearchonline/Fulltext/1968/07000/The_Discovery_of_Grounded_Theory__Strategies_for.14.aspx

17. Gomez GB, Eakle R, Mbogua J, Akpomiemie G, Venter WDF, Rees H. Treatment And Prevention for female Sex workers in South Africa: protocol for the TAPS Demonstration Project. BMJ Open. 2016 Sep 1;6(9):e011595.

18. Sibanyoni M. Sex in the Cities: Comprehensive healthcare for sex workers. In 2015. Available from: http://www.saaids.co.za/Presentations2/Wednesday%2010%20June%202015/Hall%206/Setellite%20session%2014h00_Wits%20RHI/pdf/SWMC%20SAAIDS%20Presentation.pdf

19. Braun V, Clarke V. Using thematic analysis in psychology. Qual Res Psychol. 2006 Jan;3(2):77–101.

20. Eakle R, Manthata G, Stadler J, Mbogua J, Sibanyoni M, Venter WDF, et al. Preparing for PrEP & Immediate Treatment: Focus Group Discussions in Advance of a Demonstration Project in South Africa. In Cape Town, South Africa; 2014 [cited 2016 Jun 22]. Available from: http://online.liebertpub.com/doi/full/10.1089/aid.2014.5606.abstract

21. UNAIDS. Sex workers' hopes and fears for HIV pre-exposure prophylaxis: recommendations from a UNAIDS consultation meeting. Johannesburg, South Africa: Wits RHI; 2014.

22. AVAC. Engagement For Impact - Home [Internet]. [cited 2017 May 4]. Available from: https://www.engagementforimpact.org/toolkit/

23. World Health Organization, United Nations Population Fund, Joint United Nations Programme on HIV/AIDS, Global Network, of Sex Work Projects, The World Bank. Implementing comprehensive HIV/STI programmes with sex workers: practical approaches from collaborative interventions. Geneva: World Health Organization; 2013.

24. Coleman J, Bohlin KC, Thorson A, Black V, Mechael P, Mangxaba J, et al. Effectiveness of an SMS-based maternal mHealth intervention to improve clinical outcomes of HIV-positive pregnant women. AIDS Care. 2017 Jan 20;1–8.

25. Robyn Eakle, Adam Bourne, Judie Mbogua, Nyaradzo Mutanha, Helen Rees. Exploring acceptability of PrEP and early treatment among female sex workers in advance of the TAPS Demonstration Project. submission under review;

For peer review only - http://bmjopen.bmj.com/site/about/guidelines.xhtml

Figure 1. The formative research process and timeline to design the PrEP and early ART interventions for TAPS.

Abbreviations: ppts = participants; SMS = Short Message System; FGDs = Focus Group Discussions

For peer review only

For peer review only - http://bmjopen.bmj.com/site/about/guidelines.xhtml

Figure 1

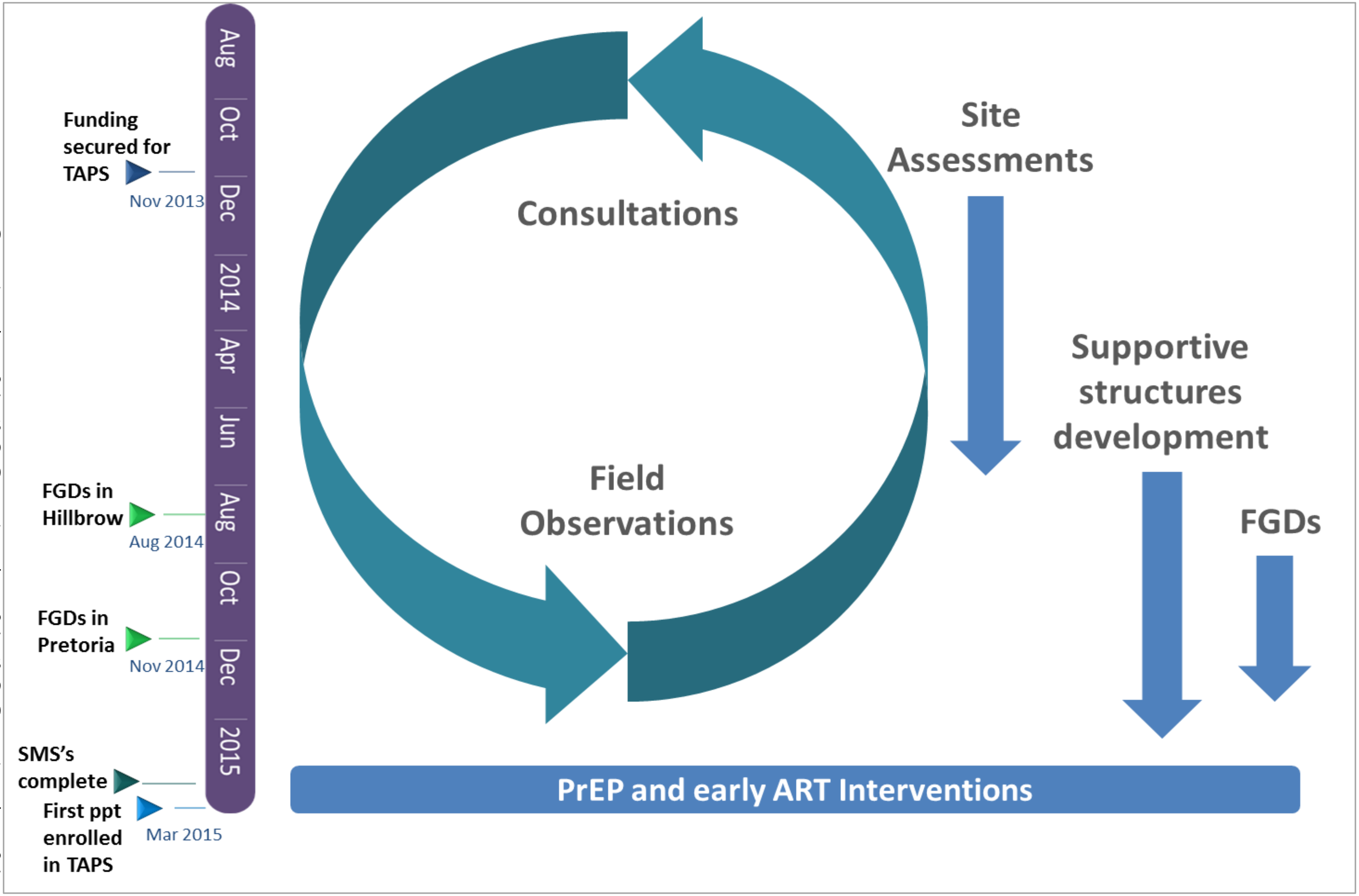
Aug
Oct
Dec
2014
Apr
Jun
Aug
Oct
Dec
2015
Funding secured for TAPS
Nov 2013
FGDs in Hillbrow
Aug 2014
FGDs in Pretoria
Nov 2014
SMS's complete
First ppt enrolled in TAPS
Mar 2015
Consultations
Field Observations
Site Assessments
Supportive structures development
FGDs
PrEP and early ART Interventions

BMJ Open

Designing PrEP and early HIV treatment interventions for implementation among female sex workers in South Africa: developing and learning from a formative research process

Journal:	*BMJ Open*
Manuscript ID	bmjopen-2017-019292.R1
Article Type:	Research
Date Submitted by the Author:	28-Feb-2018
Complete List of Authors:	Eakle, Robyn; Wits Reproductive Health and HIV Institute, ; London School of Hygiene and Tropical Medicine, Social and Environmental Health Research<br>Mutanha, Nyaradzo; Wits Reproductive Health and HIV Institute<br>Mbogua, Judie<br>Sibanyoni, Maria ; Wits Reproductive Health and HIV Institute<br>Bourne, Adam; La Trobe University, Australian Research Centre for Sex, Health & Society<br>Gomez, Gabriela; London School of Hygiene and Tropical Medicine,<br>Venter, WD Francois; University of the Witwatersrand, Wits RHI<br>Rees, Helen; University of the Witwatersrand, Wits RHI
**Primary Subject Heading**:	HIV/AIDS
Secondary Subject Heading:	Health services research, Public health, Qualitative research, Research methods
Keywords:	Sex work, HIV prevention, pre-exposure prophylaxis, formative research

SCHOLARONE™
Manuscripts

For peer review only - http://bmjopen.bmj.com/site/about/guidelines.xhtml

Designing PrEP and early HIV treatment interventions for implementation among female sex workers in South Africa: developing and learning from a formative research process

Robyn Eakle*[1, 2,3], Nyaradzo Mutanha[1], Judie Mbogua[1], Maria Sibanyoni[1], Adam Bourne[4], Gabriela Gomez[1, 2], Francois Venter[1], Helen Rees[1]

[1]Wits Reproductive Health and HIV Institute, Faculty of Health Sciences, University of the Witwatersrand, Johannesburg, South Africa
[2] Social and Mathematical Epidemiology (SaME), Department of Global Health and Development London School of Hygiene and Tropical Medicine, UK
[3] Sigma Research, Department of Social and Environmental Health Research, London School of Hygiene and Tropical Medicine, UK
[4] Australian Research Centre for Sex, Health & Society, La Trobe University, Melbourne, Australia.

*Corresponding author:
Robyn Eakle
Wits Reproductive Health & HIV Institute
University of the Witwatersrand,
Hillbrow Health Precinct
22 Esselen Street
Hillbrow, 2001
Johannesburg
South Africa
e: reakle@wrhi.ac.za

Author emails:

Robyn Eakle	robyn.eakle@lshtm.ac.uk; reakle@wrhi.ac.za
Nyaradzo Mutanha	missnyarie@gmail.com
Judie Mbogua	judie.mbogua@gmail.com
Maria Sibanyoni	msibanyoni@wrhi.ac.za
Adam Bourne	A.Bourne@latrobe.edu.au
Gabriela Gomez	Gabriela.Gomez@lshtm.ac.uk
Francois Venter	fventer@wrhi.ac.za
Helen Rees	hrees@wrhi.ac.za

Word count: 5,748
Number of figures/tables: 3
References: 37

Key words: HIV prevention, biomedical prevention products, pre-exposure prophylaxis (PrEP), sex work, formative research

For peer review only - http://bmjopen.bmj.com/site/about/guidelines.xhtml

Abstract

Objectives

The objective of this research was to design relevant, tailored oral pre-exposure prophylaxis (PrEP) and early antiretroviral (ART) interventions for female sex workers (FSWs) in South Africa. This paper examines the methods, process, and outcomes of employing an inductive approach to formative research exploring intervention feasibility and acceptability.

Setting

Research was conducted in several sex work-related settings including five sites in and around clinics, and stakeholder offices.

Participants

Participants in this research included stakeholders, experts in the field, and FSWs. This included at least 25 separate engagements, 14 local organizations, and eight focus group discussions (FGDs) with 69 participants, in addition to ad hoc meetings.

Results

The first set of outcomes consisted of five selected methods: 1) stakeholder consultations; 2) site assessments and selection; 3) field observations and mapping; 4) development of supportive structures to encourage retention and intervention adherence; 5) FGDs conducted with FSWs to explore specifics of acceptability. In terms of feasibility, two sites were selected in central Johannesburg and Pretoria out of five considered. The urban site contexts varied, necessitating adjustments to intervention implementation. There was overall support for PrEP and early ART from stakeholders and FSWs. Concerns included potential issues with adherence to PrEP (and early ART), possible reduction in condom use, resistance to ARVs, and burden on scarce resources. These concerns indicated where special attention should be focused on education, messaging, and programming, as well as development of supportive structures.

Conclusions

The inductive approach allowed for a wide range of perspectives, defining population needs and accessibility. This research illustrated how similar sex work environments can vary and how implementation of interventions may not be uniform across contexts. Lessons learned in details could assist in future project designs and implementation of new interventions where feasibility, social and cultural factors affecting acceptability must be considered.

Strengths and Limitations

- This formative research process drew on principles of grounded theory allowing for an inductive, iterative approach to drive the selection of the most appropriate methods for gathering a broad spectrum of data aimed at intervention design.
- Five methods were selected, providing an array of data for decision making and intervention design.
- The final selected project sites were chosen for their nearer uniformity than for diversity; results may not translate beyond this study, however lessons learned will be applicable.

For peer review only - http://bmjopen.bmj.com/site/about/guidelines.xhtml

Introduction

Several guidance documents highlight the need for formative research both when preparing for larger studies and to design the implementation of new public health interventions (1,2). Formative research includes the assessment of feasibility, reach, acceptability, and need of populations to strengthen planned uptake and use of interventions. In particular, formative research aims to ensure the capacity for physical implementation and responsiveness to cultural, social, economic and physical environments (3–5). However, this phase of work is frequently not reported in detail and important lessons learned may be lost. This paper describes the detailed decision making and conduct of formative research undertaken to design two new HIV prevention and treatment interventions delivered to female sex workers (FSWs) in a demonstration project in South Africa.

Oral pre-exposure prophylaxis (PrEP) using antiretroviral (ARV) drugs given to HIV-negative individuals to prevent HIV infection, has been shown to be efficacious in multiple clinical trials (6). In addition, HIV treatment can be given to HIV-positive people as soon as they are diagnosed, called test and treat or early antiretroviral treatment (ART). PrEP and early ART are now recommended in the standard of HIV care by the World Health Organization (WHO) (7). These two interventions have also been listed as priorities for operational research. When the study described here was in its infancy, PrEP and early ART were being considered for potential integration into the standard of care in South Africa, which still experiences one of the world's largest HIV epidemics (8). While South Africa has a generalised epidemic, key populations at highest risk for HIV, such as sex workers, have been in need of prioritised and tailored HIV prevention, treatment and care (9).

Demonstration projects were recommended by the WHO in 2013 to answer implementation questions relating to feasibility and acceptability of oral PrEP (10). The call prioritised research for key populations such as sex workers, who have been shown by mathematical modelling to be ideal candidates for PrEP, especially in combination with early antiretroviral (ART) treatment for HIV-positive people (11–13). In the previous decade, HIV prevalence among female sex workers in South Africa was found to be between 46% and 69% (14–16), with recent research estimating a prevalence of 72% in the greater Johannesburg area (17).

Before pursuing implementation research on PrEP and early ART delivery in South Africa, formative research was needed to first shape the interventions. The research presented here employed a comprehensive and inductive approach to formative research that explored feasibility and acceptability of PrEP and early ART, with a view to informing and executing the targeted interventions. This work formed the basis for the design of the TAPS (Treatment And Prevention for Sex workers) Demonstration Project, the purpose of which was to demonstrate how these two interventions could be implemented among FSWs, and inform national scale-up in South Africa (18). We explore and illustrate the approach and process undertaken to define and carry out the formative research, describe how the results informed the overall design of the oral PrEP and early ART interventions for TAPS, and reflect on challenges and successes encountered during the process.

For peer review only - http://bmjopen.bmj.com/site/about/guidelines.xhtml

Methods

This formative research took place between August 2013 and March 2015, and employed an inductive approach based on the principles of grounded theory (19). An inductive approach to data collection is iterative in nature and, rather than seeking to test a hypothesis or assumption, allows findings to emerge from the bottom up. Findings from one stage of data collection can inform decisions about the selection of subsequent methods and identify new, perhaps unanticipated avenues for data collection to help address the key research questions. Grounded Theory (20) is a qualitative research approach that operates inductively and can be used to guide robust data collection and analysis in an iterative manner.

Our overall, initial goal was to implement and evaluate oral PrEP and early ART interventions in the TAPS project integrated into a pre-defined service delivery programme for FSWs. To arrive at this goal we faced several important questions to address in the formative phase, including:

1) was there support from stakeholders to test the implementation of PrEP and early ART among FSWs;
2) where could/should the interventions be delivered, how, and by whom;
3) how should FSWs be engaged in the project (both in the formative phase and in the active study);
4) what structures should be included to support delivery; and,
5) how did women conceive of acceptability as users of the interventions (and therefore affect demand)?

Since formative research can take many forms including exploring feasibility of supportive structures and logistics for physical delivery of the intervention, as well as exploring the acceptability among populations in different contexts (2), it was important to use an inductive approach to selecting methods driven by the above questions and findings as they emerged. At the beginning of this process, there was a wide original scope within which to consider a large range of logistical possibilities (e.g. site locations, adherence support structures, community capacity for involvement) for the design and implementation of the interventions, also an important reason not to pre-define all methods which would have limited the scope of design consideration.

Additionally, at the start of the formative work, we knew that the overall aims of the larger TAPS Demonstration Project would be to explore whether FSWs will take up early ART or PrEP, whether the service delivery mechanism was capable of handling the increase in resource needs that might be required, and what the implications of the implementation of these interventions might be, including overall costs should the interventions be scaled up (18). TAPS was part of the Wits RHI Sex Worker Programme (SWP), a comprehensive health and well-being programme for sex workers running for over 20 years in Johannesburg and other provinces in South Africa (21). In this regard, the main purpose of the formative research was to develop interventions which had the best possible potential for success as well as for evaluation. This premise drove the formative research process, and early on defined the imperative to ensure that sex workers were included throughout.

Decision making about which methods, sites, and stakeholders with whom to engage at each step was guided by feedback from community members and the data collected. Principles from the Good

For peer review only - http://bmjopen.bmj.com/site/about/guidelines.xhtml

Participatory Practice Guidelines (GPP) developed by UNAIDS and AVAC were also followed, promoting multi-level stakeholder engagement as a core component of research (1). In line with these guidelines, a range of stakeholders were engaged, including sex workers, sex work related organizations, and the Department of Health (DoH), further described in the results section.

The selected research activities were chosen according to the evolving data and eventually fell into five core categories of methods: consultations, site assessments, field observations and mapping, development of supportive structures and messaging, and FDGs. These methods generated three primary sources of data which informed the design of the interventions: 1) recorded minutes and reports of meetings with stakeholders; 2) notes from field observations collected during engagement with sex worker communities and the environments within which they work, as well as the process of hotspot mapping, experiences at potential clinic sites, and through the development of supportive structures; and, 3) transcripts of FGDs. Outcomes included the chosen formative research methods, the lessons learned from data collection, and the final design of the interventions as well as relevant data collection and monitoring tools and supportive structures. These data were collected by a combination of researchers, sex worker peer educators, and clinical staff.

Data analysis

Data were continuously collected and analysed using an inductive, Grounded Theory approach over 18 months. Field notes, meeting notes, written reports, and FGD transcripts, were reviewed as activities occurred to identify key themes for further exploration and to define next steps. In this regard, this approach employed a continuous review of data collected in a rolling fashion where the researchers would note significant pivot points in learning to then decide on the next step in research until final decisions on project design aspects had been made. For example, the decision to disqualify a site could be made due to low accessibility of the population only discovered during onsite exploration, and then that decision would be immediate and final leading to more attention paid to the next step at other sites. Site selection and staff recruitment represented the end of the first major phase of formative research. Community mobilization was then focused at the selected sites where testing of messaging, development of supportive systems, and development and testing of potential data collection tools continued led by clinic staff, peer educators, and potential end users.

FGDs were conducted at each of the final selected sites in multiple languages to suit the participants and analysed following principles of thematic analysis (22), concentrating on themes originating from the field research incorporated in the FGD guides as well as allowing for organic themes to emerge during the discussions. Further details of the methods and results from the FGDs are presented in a companion paper (23).

Results

As this is primarily a methods paper, results are presented in five sections according to the chosen methods as products of the grounded/inductive approach. Sections also relate to the questions addressed by the methods, how the methods were undertaken, and lessons learned influencing the design and execution of the interventions.

For peer review only - http://bmjopen.bmj.com/site/about/guidelines.xhtml

These five methods were: 1) stakeholder consultations; 2) site assessments and selection; 3) field observations and mapping; 4) development of supportive structures to encourage retention and intervention adherence; 5) FGDs conducted with potential end-users to explore specifics of acceptability. These are presented in Table 1 which maps methods to the research questions and data types.

Additionally, a diagram of the methods and the timeline during which they occurred is shown in Figure 1. This figure illustrates how some activities were continuous and others discrete, as research is very rarely a clean, linear process and in this case the data were continually informing the decision making.

Table 1. Formative research methods and their attributes mapped to research questions

Research Question	Method(s)	Data Type	Stage/Aspect in process
1. Was there support from stakeholders to test the implementation of PrEP and early ART among FSWs?	Consultations, Site Assessments, FGDs	Meeting reports and minutes, field notes, FGD transcripts	First step, continuous throughout process
2. Where could/should the interventions be delivered, how, and by whom?	Consultations, Site Assessments, Field Observations	Meeting reports and minutes, field notes	Second step, continuous until sites finalized
3. How should FSWs be engaged in the project (both in the formative phase and in the active study)?	Consultations, Field Observations	Meeting reports and minutes, field notes	Incorporated across all steps
4. What structures should be included to support delivery?	Consultations, Site Assessments, Field Observations, Development of supportive structures, FGDs	Meeting reports and minutes, field notes, FGD transcripts	Concluded in later steps of the process, but taken from lessons learned throughout
5. How did women conceive of acceptability as users of the interventions (and therefore affect potential demand)?	Consultations, Site Assessments, Field Observations, FGDs	Meeting reports and minutes, field notes, FGD transcripts	A component of early phases, but more discretely determined in final steps

[Insert Figure 1. Formative Research Process Graphic]

Stakeholder Consultations

This method sought to answer the question relating to support for the TAPS interventions from stakeholders, but also resulted in addressing some of the other questions around engaging FSWs, potential locations for the research sites, and concepts of acceptability. Research began with this activity in order to ascertain a baseline of stakeholder knowledge of and attitudes towards PrEP and early ART, as well as attitudes towards sex workers, which denoted initial levels of acceptability for the interventions in this population. We initially conducted three community consultations in 2013 with a total of 81 attendees from sex worker communities and partner organizations in Hillbrow and

For peer review only - http://bmjopen.bmj.com/site/about/guidelines.xhtml

Ngodwana, where a new sex worker clinic was in the initiation phase. Additionally, an international consultation of 38 attendees was held in Hillbrow in 2013 and included representatives from sex work communities and organizations, funders, UNAIDS and WHO (24). These consultations allowed for mapping of organizations and individuals from which to move into the next phase of consultations and formative research.

Smaller meetings were held with local DoH representatives in Johannesburg, Mpumalanga, Phongolo, and Pretoria to develop partnerships and potential support agreements for the TAPS project. Updates on, and engagement with, the plans for the TAPS interventions continued at quarterly and other scheduled meetings with local DoH and partners. Parallel meetings were also held with National DoH and the South African National AIDS Council (SANAC). These meetings also informed the site selection in terms of support and local capacity.

Sex workers at each potential site, driven largely by peer educators, helped to identify community organizations to engage for additional perspectives. Stakeholder engagement, both formal and informal, was tracked using an engagement tracking tool (25) developed by the HIV prevention advocacy group, AVAC. Separate meetings were held with 14 different organizations, as well as a number of sensitization trainings provided by the TAPS team and SWEAT to local providers and DoH officials. These added up to at least 25 meetings; however many additional ad hoc, less formal engagement occurred as well during this time which was not possible to definitively quantify.

Feedback from the consultations pointed to varying degrees of support and acceptability depending on local capacity to take on new interventions and scepticism towards the interventions, and particularly PrEP. Stakeholders affirmed that implementing PrEP and early ART together would promote synergistic delivery, as well as potentially normalize the use of ARVs in providing options to both HIV-negative and positive sex workers. However, concerns were voiced about adherence to PrEP (and sometimes early ART), as well as the potential for reduction in condom use, increases in risk of resistance to ARVs, and burden on scarce resources. These concerns indicated where special attention should be focused, such as providing evidence about possible resistance to PrEP and strategies for and the philosophy around supporting adherence and condom use, both in early messaging and education for sex workers, implementing and policy partners, as well as where to focus messaging and monitoring when implementing the interventions in TAPS.

Finally, consultations were also used to identify potential community advisory board (CAB) members. Meetings were held with individuals expressing interest in participation to inform them about the study and the CAB. These discussions continued until a CAB with up to 12 members (set as the limit for the group which might fluctuate in attendance over time) was established. The CAB incorporated representatives from the local police force, sex worker advocacy organizations, sex workers themselves, and religious organizations. A total of five CAB meetings were held before TAPS was launched in March 2015. The CAB became both a result of the formative research and another source of consultation. The CAB also functioned as a supportive structure as members helped spread knowledge of the interventions and the TAPS project, as well as lobby for expansion of the interventions to more sites, populations, and organizations.

Site assessments and selection

For peer review only - http://bmjopen.bmj.com/site/about/guidelines.xhtml

Site selection primarily addressed the question of where the project could/should be implemented, and much was learned in this process which is further reported in the field observations. Finalizing site selection was a critical step in moving forward with the design of the interventions. We aimed to select 2-3 sites (according to funding and capacity for managerial oversight) with the following population criteria: access to a large number of FSWs (>200 according to early sample size calculations (18)), populations with a relatively balanced proportion of HIV-negative and HIV-positive women, and accessibility of clinics. Physical feasibility of the clinics to implement the interventions was assessed through reviews of space, clinical and laboratory infrastructure, required site permissions and approvals, and supply chain mechanisms. Support for the interventions was explored with the local DoH and sex worker communities, as well as identification of logistical gaps. Site visits, hot spot mapping, and site assessments were conducted to determine the feasibility of delivering PrEP and early ART.

Assessments were conducted in four of the original nine Wits RHI SWP sites plus Pretoria as a new site. The other five sites did not meet initial criteria (e.g. size of FSW population, clinic feasibility). These five sites featured existing SWP clinics already delivering ARVs per national guidelines or had plans to implement new clinics, interest and support from the local communities, and potential access to a large group of FSWs in areas where HIV prevalence was high. This information came from the initial consultations and community engagement and mapping, as well as internal programme data. These sites were located in: Ngodwana (rural village in Mpumalanga province), Phongolo (rural trucking site in KwaZulu Natal province), Hillbrow (central, inner-city Johannesburg), City Deep (peri-urban trucking site immediately south of Johannesburg), and the Pretoria central business district (CBD), the latter three of which were located in Gauteng province. Figure 2 illustrates the sites situated across several provinces in South Africa.

[Insert Figure 2. Map of TAPS and Wits RHI SWP Sites in South Africa]

Following the site assessments, three sites were eliminated. Ngodwana was eliminated due to lack of infrastructure and building delays, as well as lack of local support. Although there was initial interest, the implementation of the interventions potentially conflicted with limited resources and competing priorities in the area. The rural sex worker community in that location do not self-identify as sex workers which would have created challenges in being able to compare formal sex workers who self-identify and those who do not as part of project evaluation. The prevalence of HIV in Ngodwana was estimated locally to be around 75-80% in a relatively small village of <300 people mostly made up of sex workers, which also would have made it difficult to implement PrEP.

City Deep was eliminated due to low clinic attendance, as well as the highly transient nature of the sex worker populations. Most of the women attending that clinic arrived in the trucks coming from all over South Africa as well as neighbouring countries, which would have made commitment to regular, repeat clinic visits difficult. The Phongolo site, located next to the Swaziland border, was also eliminated as women tended to move in and out of the town and were away for as many as three to four months at a time.

As a result, the clinics in Hillbrow and Pretoria were selected as the two TAPS sites. Hillbrow was chosen because of the long-standing Wits RHI sex worker clinic which maintains strong ties to a large

For peer review only - http://bmjopen.bmj.com/site/about/guidelines.xhtml

community of sex workers and their managers (brothel owners, 'pimps'). At the time, HIV prevalence was estimated locally at around 50%. The clinic is situated in an area with a high concentration of brothels and street based locations within walking distance, but also with a number of surrounding outreach areas in the Johannesburg CBD, Yeoville, Jeppe, and Rosettenville. For these reasons, this site was considered "low-hanging fruit" for new intervention implementation, due to the substantial population of women at risk of HIV infection and as a location with solid community support.

In Pretoria, tailored sex worker services and relationships with the community were new at the Sediba Hope clinic in the heart of the CBD, however there was strong support from the local DoH and expressed need from the sex workers themselves. The newness of such interventions in the area posed a challenge as to whether PrEP and early ART could be implemented within a new sex worker clinic in a new setting, and what it would take to do so, however this was determined to be a potential strength (and weakness) for implementing these new interventions in a new clinic and the lessons that could be learned.

Field Observations and Mapping

Field observations and mapping elicited information relating to the how of intervention implementation, engaging the community, and acceptability. Data were collected through a variety of engagements in the field centring on community mobilization activities, accruing organically in scope, as discussions and interactions within the communities pointed to additional contacts for engagement. Each point of contact presented opportunities for new consultations and/or locations to conduct outreach. Feedback from the community about potentially offering PrEP and early ART was collected during peer and clinical staff outreach and workshops, which also aimed to elicit values related to acceptability and ultimately generate demand for the interventions.

The core of the community mobilization and exploration work was conducted through outreach. In this case, outreach consisted of health education, condom promotion use, and a spectrum of services provision from HIV testing and counselling to full clinical services in mobile units in locations accessible to the population (26). TAPS staff accompanied staff and peer educators from the SWP during their normal outreach activities to probe the local sex worker populations for any knowledge or perceptions of PrEP and early ART, as well as start to introduce related information. This resulted in learning more about where and how sex workers operated and how they interacted with health services, informing viability of given sites. We found high interest in and a general cursory understanding of the interventions, particularly in the Johannesburg Hillbrow area where some women had been previously engaged in research studies around PrEP and microbicdes. These engagements led us to further think about which supportive structures would be needed and how messages would have to define the differences between PrEP and early ART. Additionally, as core feature of the SWP, interventions were designed around outreach which was identified as a central aspect critical to generating demand and recruiting participants for TAPS.

Hot spot mapping was conducted during outreach to determine the number of FSWs working near the potential sites as well as their working hours which would allow us to focus community education and recruitment efforts. The methods utilized and built on the results produced by the South African Health Monitoring Study (SAHMS), which drew on multiple mapping methods

For peer review only - http://bmjopen.bmj.com/site/about/guidelines.xhtml

including time-location and the "wisdom-of-crowds" (17). This activity in combination with prior experience derived through the SWP, revealed that time and location of sex work is driven largely by client availability. Peak working hours were recorded in each site as critical data dictating when women could be free to attend the clinics. These varied dramatically from site to site, where working hours may begin at 10 am in some places, and much later in the afternoon in others. Local languages at each site were also documented so that study materials could be translated and appropriate staff hired.

One of the most important aspects of learning was around the sex work environment, which was somewhat known from our engagement with the larger SWP, but important for staff to see and understand first hand. We assumed, according to previous knowledge, three general categories of sex work locales within the two urban settings. Brothels or hotels usually offer alcohol and entertainment, including combinations of music, dancing, and bar games. Many are former or existing hotels while others are simply bars with backrooms. Security protecting sex workers from violent clients is common in these establishments. Secondly, street-based sex work occurs in high traffic areas where sex workers pick up clients, and either join them in vehicles or have sex in a nearby location such as an alley. The third category comprises the "dark places" (IsiZulu: *mnyamandawo*), informal locations in empty lots or other uninhabited/unoccupied buildings or areas where sex workers build rudimentary structures in which to work.

However, in both cities there is also a fourth, more hidden sex work market found online. Awareness of the online market was generated through conversations with sex workers during outreach and through peer educators, some of whom had online pages and knew of others with similar arrangements. Sex work conducted online usually involves regular clients met through a web-based connection, where business is conducted out of private spaces (personal homes, private spaces run by an owner, upscale hotels or clubs). This population was engaged later in the process as they are less visible to implementers and researchers, and difficult to build relationships with given their desire for anonymity.

Finally, earning ability, directly associated with the type of sex work locale, was identified as an important aspect of being able to take time off to come to the clinic even for short periods of time. Sex workers operating in brothels tend to be able to charge higher rates for sex, usually between R50 and R150 (about 3-10 USD), depending on the status of the brothel and the cost of room rental incurred by the sex workers. On the street, rates are around R50, and in a dark place sex can be R20-30 (about 1.30-2 USD). Making less money meant more difficulty in taking time away from work to attend the clinic for regular PrEP or treatment related appointments.

Development of supportive structures and messaging

During consultations, a number of stakeholders provided views on possible components to be incorporated in the intervention design, in particular supportive structures. Supportive structures are generally anything outside of the standard of care which act to promote intervention uptake and use. Mechanisms for supporting intervention adherence and retention in care were explored, however we decided only to consider those which could be incorporated into a national programme. Among the possibilities were the use of MEMS caps (electronic bottle caps which would count bottle openings as a proxy for pill withdrawals and thus, adherence), pill counts (as conducted in clinical

For peer review only - http://bmjopen.bmj.com/site/about/guidelines.xhtml

trials), and mHealth solutions in the form of short message service (SMS) messages. After discussing options with DoH partners, it was concluded that SMS, as demonstrated by the ongoing MAMA Connect project (27), could be scaled up in a national programme, whereas other options would not be feasible.

A participatory process led by representatives of RHI's mHealth and Mental Health teams was undertaken through a series of workshops where themes around healthy living were developed followed by relevant SMS messages by peer educators. Both male and female sex worker peer educators worked in groups to create the messages which aimed to encourage healthy choices and wellness. These messages took into account the importance of avoiding inadvertent disclosure of HIV status to non-participants, and could still be used by the SWP after TAPS concluded. This process produced 110 SMS messages which would be sent once a week in succession to all participants who signed up for the service.

Experiences from early outreach activities directly influenced the messaging employed in each location, as well as educational materials used as part of supportive structures for generating demand and promoting awareness of the interventions. Messages focused on defining the interventions, addressing common myths and concerns around side effects, and providing information on efficacy and access.

Peer educator and potential user feedback indicated that personal testimonies are highly valued by women in both locations, so these were included on the informational pamphlets. Trained peer educators also enrolled in the study were invited to become ambassadors for the interventions so that they could directly relate their experiences and dispel rumours about side effects.

Finally, additional supportive structures for the interventions and the women using them included the CAB consisting of sex work related agencies who could help women with legal issues or post-rape care who were then also aware of the TAPS project. These helped to build on the existing SWP tried and tested referral systems where we could ensure women would get additional support beyond the scope of the TAPS clinics as needed. One result of the consultations, as well, was the need for holistic sensitivity training around sex work which was conducted with every staff member, from cleaner to clinician, at both sites. Although a more passive support mechanism provided by a local community partner organisation, this was an important, supportive measure in ensuring women felt welcome at the clinics.

Focus Group Discussions

The FGDs were the culmination of the formative research exploring acceptability of PrEP and early ART to be delivered in the two clinic sites. Four FGDs were held in each site with a total of 69 participants. The FGDs comprised important final steps in informing design as they explored a more focused community perspective of intervention acceptability on two main levels. One level consisted of data concerning logistics and preferences around physical delivery (location, preferred clinic times, frequency of visits and HIV testing). This also spoke to the "whom" of service delivery were a significant component of the discussions was the importance of having sensitized nurses and other staff as well as underscoring the best practice of peer-driven education and communication. The other level included social and structural aspects where elements of stigma and socioeconomic

For peer review only - http://bmjopen.bmj.com/site/about/guidelines.xhtml

norms (e.g. where the need for income might supersede health) that might affect uptake and use of PrEP and early ART were explored. Since this paper is focused on feasibility and early stages of acceptability, the end-stage formative data from the FGDs are presented in a companion paper (23).

Discussion

In this paper, we have described in detail the formative research process and findings used to inform the design of the PrEP and early ART interventions implemented in TAPS. The grounded, inductive approach afforded a breadth of information around potential site locations, as well as community and stakeholder perspectives, including potential opportunities barriers to successful implementation and nuanced aspects of the urban sex work settings. These aspects were critical in considering feasibility of implementation and how to ensure the interventions accomplished reach, accessibility, acceptability, and filled the needs of potential end-users. This process allowed for consolidation of a broad scope of lessons learned to inform intervention design.

Feasibility played a significant role in early decision making as to whether PrEP and early ART could be implemented, and acceptability from stakeholders, including sex workers themselves, ran in parallel. Questions of feasibility addressed site capacity, experience in delivering ARVs, experience with and access to FSWs, site locations related to FSW populations, and existing supply chains. Early acceptability was determined through consultations and engagement with potential end users in the community during outreach. Factors influencing feasibility and acceptability would not have been as comprehensively understood, such as the day to day clinic operation and the physical contexts and locales of sex work, without repeated site visits and time spent in the field. Continuous, in-depth interactions with the women themselves allowed the TAPS team to better understand sex workers' needs in addition to how interventions reach could be maximized. Additionally, identifying and addressing potential biases from providers and other stakeholders in the provision of the interventions was essential to avoid issues with maintaining permissions and supply chains as well as supportive services.

Generally, it would be expected that sex work populations and industries, with similar urban contexts and in relative geographical proximity, would be the same in both places and that interventions could be implemented uniformly in both locations. However, the formative research demonstrated just the opposite. While there were some similarities in how women live and work in terms of the types of locales and spaces, there were also significant differences. Sex work locales can be similarly categorized in the two cities, but the organization of these spaces and the make-up of them within Hillbrow and Pretoria is quite different in terms of women's personal safety and earning capability, for example. This translates into different sex worker populations and market dynamics and the need for adaptations to intervention design. Outreach strategies (groups versus one on one discussions) and messaging channels (word of mouth, top down through brothel owners and pimps, or developing online contacts) were dictated directly by context and the expressed needs of the FSWs.

While many projects have most likely undertaken similar processes in designing interventions for evaluation, taking an explicit inductive approach and the subsequent outcomes have not been reported. For instance, the Microbicides Development Programme (MDP) has 102 associated references (28), the majority of which come from the MDP 301 study (29). There is a detailed

For peer review only - http://bmjopen.bmj.com/site/about/guidelines.xhtml

published study protocol (30), and several background papers (31–36), however, no detail on the qualitative aspects contributing to the design-related decision making. By presenting this paper, we argue that those details are important for future study, intervention, and product implementation design where previous lessons learned from formative research and methodology can potentially support future researchers and implementers as well as inform research processes and design.

Conclusions

The lessons learned from this formative research process were directly applied to the design and implementation of the PrEP and early ART interventions delivered through the TAPS project. The inductive approach afforded the opportunity to adapt and include voices and perspectives, which might have otherwise been missed, and clarified the needs of the population as well as how to reach them appropriately. This research illustrated how sex work environments can vary, even when the settings are very similar, and therefore implementation of interventions is unlikely to be uniform across contexts. Formative research is critical in designing interventions, especially in new environments but also in well-known contexts. Including intensive stakeholder engagement in formative research will help to ensure that interventions are designed with feasibility and relevance for populations in mind.

Declarations

Ethics approval and consent to participate

Formal ethical approval was provided by the Wits Human Ethics Research Committee (reference number: M131009) for the focus group discussions which included information testing and community engagement in the field to inform the development of discussion guides. All documents containing data were saved electronically in central folders with limited access to relevant project staff. No participant identifiers were included in any of the reports that were produced. With regard to other formative research elements, no individual data were collected or reported, and ethical principles of the Helsinki Declaration were strictly adhered to as part of this iterative and flexible approach.

Availability of data and materials

All data generated or analysed during this study are included in this published article.

Competing interests

The authors have no declarations or conflicts of interest associated with this work.

Funding

This research was in part supported by funds from AIDS Fonds Netherlands (and the Bill and Melinda Gates Foundation (grant OPP1084416).

Patient and Public Involvement

This research was entirely based on potential patients' priorities, experience, and preferences. Both potential end-users and other stakeholders were involved throughout each step of the formative research and in the active TAPS project as well, as described throughout this paper. The Wits RHI SWP is based on a sex worker peer-led model of service which supported all of this work (including

For peer review only - http://bmjopen.bmj.com/site/about/guidelines.xhtml

general outreach as well as recruitment for the FGDs). Results of this work were disseminated to the community and stakeholders as decisions were made and through the implementation of TAPS.

Author Contributions

RE developed the research agenda, designed data collection tools, participated in data collection, wrote and collected field notes, analysed the data, and drafted the paper. JM participated in data collection and analysed data. NM and MS participated in data collection. AB supported design of data collection tools and data analysis. RE, GG, FV, and HR made final decisions on intervention design. All authors reviewed and contributed to the paper.

Acknowledgements

We would like to thank the many women who participated in this formative research as well as the stakeholders and staff who engaged in helping us design the PrEP and early ART interventions for the TAPS Demonstration Project.

For peer review only

For peer review only - http://bmjopen.bmj.com/site/about/guidelines.xhtml

References

1. UNAIDS. Good participatory practice: Guidelines for biomedical HIV prevention trials [Internet]. 2011 [cited 2016 Sep 28]. Available from: http://www.unaids.org/en/resources/documents/2011/20110629_JC1853_GPP_Guidelines_2011%20OK

2. Tolley EE, Ulin PR, Mack N, Succop SM, Robinson ET. Qualitative Methods in Public Health: A Field Guide for Applied Research. John Wiley & Sons; 2016. 480 p.

3. Milat AJ, King L, Bauman AE, Redman S. The concept of scalability: increasing the scale and potential adoption of health promotion interventions into policy and practice. Health Promot Int. 2013 Sep 1;28(3):285–98.

4. Milat AJ, Bauman A, Redman S. Narrative review of models and success factors for scaling up public health interventions. Implement Sci. 2015;10:113.

5. World Health Organization. Scaling up projects and initiatives for better health: from concepts to practice [Internet]. 2016 [cited 2017 Apr 17]. Available from: http://www.euro.who.int/__data/assets/pdf_file/0004/318982/Scaling-up-reports-projects-concepts-practice.pdf

6. Fonner VA, Dalglish SL, Kennedy CE, Baggaley R, O'Reilly KR, Koechlin FM, et al. Effectiveness and safety of oral HIV preexposure prophylaxis for all populations. AIDS Lond Engl. 2016 Jul 31;30(12):1973–83.

7. World Health Organization. WHO | Guideline on when to start antiretroviral therapy and on pre-exposure prophylaxis for HIV [Internet]. 2015 [cited 2015 Oct 28]. Available from: http://www.who.int/hiv/pub/guidelines/earlyrelease-arv/en/

8. UNAIDS. Global AIDS Update [Internet]. 2016. Available from: http://www.unaids.org/en/resources/documents/2016/Global-AIDS-update-2016

9. South African National AIDS Programme. South Africa's National Strategic Plan for HIV, TB, and STIs 2017-2022 [Internet]. 2017. Available from: http://sanac.org.za/wp-content/uploads/2017/05/NSP_FullDocument_FINAL.pdf

10. World Health Organization. PrEP demonstration projects: A framework for country level protocol development. A draft for review and discussion. 2013.

11. Cremin I, Alsallaq R, Dybul M, Piot P, Garnett G, Hallett TB. The new role of antiretrovirals in combination HIV prevention: a mathematical modelling analysis. AIDS. 2013 Jan;27(3):447–58.

12. Gomez GB, Borquez A, Case KK, Wheelock A, Vassall A, Hankins C. The Cost and Impact of Scaling Up Pre-exposure Prophylaxis for HIV Prevention: A Systematic Review of Cost-Effectiveness Modelling Studies. PLoS Med. 2013 Mar 12;10(3):e1001401.

13. Hallett TB. Priorities across the continuum of care: modelling the impact of interventions on mortality and HIV transmission. In Vancouver, Canada; 2015.

14. Rees H, Beksinska ME, Dickson-Tetteh K, Ballard RC, Htun Y. Commercial sex workers in Johannesburg: risk behaviour and HIV status. South Afr J Sci. 2000 Jun;96:283–4.

For peer review only

For peer review only - http://bmjopen.bmj.com/site/about/guidelines.xhtml

15. Williams BG, Taljaard D, Campbell CM, Gouws E, Ndhlovu L, Van Dam J, et al. Changing patterns of knowledge, reported behaviour and sexually transmitted infections in a South African gold mining community. AIDS Lond Engl. 2003 Sep 26;17(14):2099–107.

16. Ramjee G, Karim SS, Sturm AW. Sexually transmitted infections among sex workers in KwaZulu-Natal, South Africa. Sex Transm Dis. 1998 Aug;25(7):346–9.

17. UCSF, Anova Health Institute, Wits RHI. South African Health Monitoring Survey (SAHMS): An Integrated Biological and Behavioural Survey among Female Sex Workers, South Africa 2013 – 2014. San Francisco: UCSF; 2015.

18. Gomez GB, Eakle R, Mbogua J, Akpomiemie G, Venter WDF, Rees H. Treatment And Prevention for female Sex workers in South Africa: protocol for the TAPS Demonstration Project. BMJ Open. 2016 Sep 1;6(9):e011595.

19. The Discovery of Grounded Theory; Strategies for Qualitative... : Nursing Research [Internet]. [cited 2014 Jun 2]. Available from: http://journals.lww.com/nursingresearchonline/Fulltext/1968/07000/The_Discovery_of_Grounded_Theory__Strategies_for.14.aspx

20. Charmaz K. Constructing Grounded Theory [Internet]. Second Edition. SAGE Publications Ltd; 2006 [cited 2018 Feb 9]. Available from: https://uk.sagepub.com/en-gb/eur/constructing-grounded-theory/book235960

21. Sibanyoni M. Sex in the Cities: Comprehensive healthcare for sex workers. In 2015. Available from: http://www.saaids.co.za/Presentations2/Wednesday%2010%20June%202015/Hall%206/Setellite%20session%2014h00_Wits%20RHI/pdf/SWMC%20SAAIDS%20Presentation.pdf

22. Braun V, Clarke V. Using thematic analysis in psychology. Qual Res Psychol. 2006 Jan;3(2):77–101.

23. Eakle R, Bourne A, Mbogua J, Mutanha N, Rees H. Exploring acceptability of oral PrEP prior to implementation among female sex workers in South Africa. J Int AIDS Soc. 2018 Feb 1;21(2):n/a-n/a.

24. UNAIDS. Sex workers' hopes and fears for HIV pre-exposure prophylaxis: recommendations from a UNAIDS consultation meeting. Johannesburg, South Africa: Wits RHI; 2014.

25. AVAC. Engagement For Impact - Home [Internet]. [cited 2017 May 4]. Available from: https://www.engagementforimpact.org/toolkit/

26. World Health Organization, United Nations Population Fund, Joint United Nations Programme on HIV/AIDS, Global Network, of Sex Work Projects, The World Bank. Implementing comprehensive HIV/STI programmes with sex workers: practical approaches from collaborative interventions. Geneva: World Health Organization; 2013.

27. Coleman J, Bohlin KC, Thorson A, Black V, Mechael P, Mangxaba J, et al. Effectiveness of an SMS-based maternal mHealth intervention to improve clinical outcomes of HIV-positive pregnant women. AIDS Care. 2017 Jan 20;1–8.

28. MDP Microbicides Development Programme [Internet]. Available from: http://www.mdp.mrc.ac.uk/publications.html

For peer review only - http://bmjopen.bmj.com/site/about/guidelines.xhtml

29. McCormack S, Ramjee G, Kamali A, Rees H, Crook AM, Gafos M, et al. PRO2000 vaginal gel for prevention of HIV-1 infection (Microbicides Development Programme 301): a phase 3, randomised, double-blind, parallel-group trial. Lancet. 2010 Oct 16;376(9749):1329–37.

30. Nunn A, McCormack S, Crook AM, Pool R, Rutterford C, Hayes R. Microbicides Development Programme: design of a phase III trial to measure the efficacy of the vaginal microbicide PRO 2000/5 for HIV prevention. Trials. 2009 Oct 27;10:99.

31. Kamali A, Byomire H, Muwonge C, Bakobaki J, Rutterford C, Okong P, et al. A randomised placebo-controlled safety and acceptability trial of PRO 2000 vaginal microbicide gel in sexually active women in Uganda. Sex Transm Infect. 2010 Jun 1;86(3):222–6.

32. Lees S, Cook C, Vallely A, Desmond N, Allen C, Kiro K, et al. Comparison of sexual behavior data collected using a coital diary and a clinic-based interview during a microbicide pilot study in Mwanza, Tanzania. Sex Transm Dis. 2010 Aug;37(8):497–501.

33. Lees S, Desmond N, Allen C, Bugeke G, Vallely A, Ross D. Sexual risk behaviour for women working in recreational venues in Mwanza, Tanzania: considerations for the acceptability and use of vaginal microbicide gels. Cult Health Sex. 2009 Aug 1;11(6):581–95.

34. MacPhail C, Terris-Prestholt F, Kumaranayake L, Ngoako P, Watts C, Rees H. Managing men: women's dilemmas about overt and covert use of barrier methods for HIV prevention. Cult Health Sex. 2009 Jun;11(5):485–97.

35. Ndinda C, Chimbwete C, McGrath N, Pool R, Mdp Group null. Community attitudes towards individuals living with HIV in rural KwaZulu-Natal, South Africa. AIDS Care. 2007 Jan;19(1):92–101.

36. Gist P, Langley D. Application of Standard Project Management Tools to Research – A Case Study from a Multi-national Clinical Trial. J Res Adm [Internet]. 2007 [cited 2018 Feb 14];XXXVIII(Number 2). Available from: https://files.eric.ed.gov/fulltext/EJ902225.pdf

For peer review only

For peer review only - http://bmjopen.bmj.com/site/about/guidelines.xhtml

Figure 1. The formative research process and timeline to design the PrEP and early ART interventions for TAPS.

Abbreviations: ppts = participants; SMS = Short Message System; FGDs = Focus Group Discussions

Figure 2. Map of TAPS and Wits RHI SWP Sites in South Africa

Note: Green labels and arrows indicate selected sites; Red labels and arrows indicate sites not selected; Grey labels and arrows indicate other Wits RHI Sex Worker Programme sites.

For peer review only

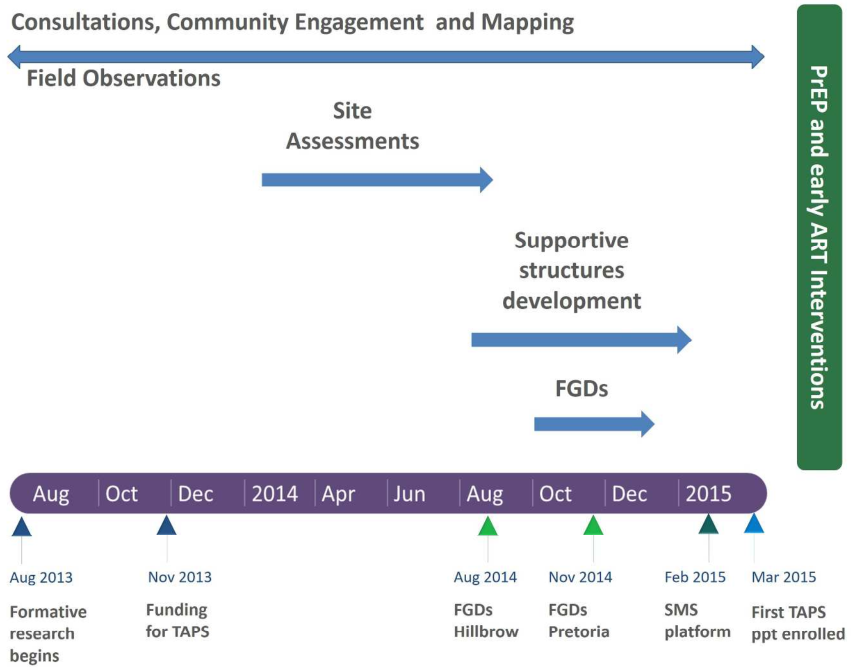

Figure 1. The formative research process and timeline to design the PrEP and early ART interventions for TAPS.

Abbreviations: ppts = participants; SMS = Short Message System; FGDs = Focus Group Discussions

243x187mm (300 x 300 DPI)

only

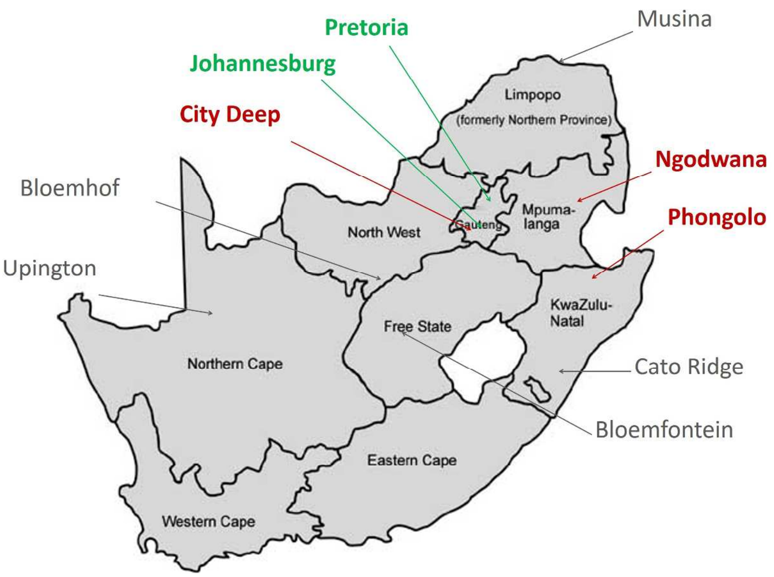

Figure 2. Map of TAPS and Wits RHI SWP Sites in South Africa

Note: Green labels and arrows indicate selected sites; Red labels and arrows indicate sites not selected; Grey labels and arrows indicate other Wits RHI Sex Worker Programme sites.

243x187mm (300 x 300 DPI)

only
